# Sex-dimorphic landing mechanics and their role within the noncontact ACL injury mechanism: evidence, limitations and directions

**DOI:** 10.1186/1758-2555-4-10

**Published:** 2012-03-15

**Authors:** Mélanie L Beaulieu, Scott G McLean

**Affiliations:** 1 School of Kinesiology, University of Michigan, Ann Arbor, MI, USA

**Keywords:** Landing, Sex-based differences, Knee, Hip, Biomechanics, ACL, Injury, In vivo

## Abstract

Anterior cruciate ligament (ACL) injuries continue to present in epidemic-like proportions, carrying significant short- and longer-term debilitative effects. With females suffering these injuries at a higher rate than males, an abundance of research focuses on delineating the sex-specific nature of the underlying injury mechanism. Examinations of sex-dimorphic lower-limb landing mechanics are common since such factors are readily screenable and modifiable. The purpose of this paper was to critically review the published literature that currently exists in this area to gain greater insight into the aetiology of ACL injuries in females and males. Using strict search criteria, 31 articles investigating sex-based differences in explicit knee and/or hip landing biomechanical variables exhibited during vertical landings were selected and subsequently examined. Study outcomes did not support the generally accepted view that significant sex-based differences exist in lower-limb landing mechanics. In fact, a lack of agreement was evident in the literature for the majority of variables examined, with no sex differences evident when consensus was reached. The one exception was that women were typically found to land with greater peak knee abduction angles than males. Considering knee abduction increases ACL loading and prospectively predicts female ACL injury risk, its contribution to sex-specific injury mechanisms and resultant injury rates seems plausible. As for the lack of consensus observed for most variables, it may arise from study-based variations in test populations and landing tasks, in conjunction with the limited ability to accurately measure lower-limb mechanics via standard motion capture methods. Regardless, laboratory-based comparisons of male and female landing mechanics do not appear sufficient to elucidate causes of injury and their potential sex-specificity. Sex-specific *in vivo *joint mechanical data, if collected accurately, may be more beneficial when used to drive models (e.g., cadaveric and computational) that can additionally quantify the resultant ACL load response. Without these steps, sex-dimorphic landing mechanics data will play a limited role in identifying the aetiology of ACL injuries in women and men.

## Introduction

Injuries to the anterior cruciate ligament (ACL) continue to occur at an alarming rate, whilst carrying significant short- and long-term morbidity and an enormous financial burden [1,2]. Individuals having ruptured their ACL, for example, have a higher susceptibility to developing, within 10-15 years of injury, osteoarthritis at the knee joint [3,4]--a degenerative joint disease that most often leads to knee arthroplasty. An additional concern is the large sex-disparity in injury rates, with females suffering noncontact ACL injuries 2-5 times more frequently than males [5,6]. Given the potentially severe outcomes of this ligament injury, research efforts remain focused on elucidating its causes. A better understanding of its aetiology will allow for more effective risk screening and prevention methods to be developed, and thus a reduction in injury rate and its associated sex disparity.

As a step toward more effective injury screening and prevention, the manoeuvres during which most ACL injuries occur have been identified and examined. Most non-contact ACL injuries are reported to arise from a sudden deceleration while either running and changing direction or landing from a jump [7]. Due to the difficult and unethical nature of deliberately provoking and analysing actual injury causing episodes, researchers have focused on obtaining joint neuromechanical data within systematic examinations of noninjurious scenarios. In particular, numerous research teams have compared the lower-limb mechanics of women performing landing tasks with those of men to gain insights into sex-dimorphic ACL injury rates [8-14]. Subsequently, those joint mechanical variables for which sex-based differences were revealed have been directly inferred as contributing risk factors for ACL injury and its associated sex-specificity. In spite of an extensive array of research with this specific intent, these variables have been inconsistently identified across studies. Such discrepancies make effective screening and prevention virtually impossible. There is a need, therefore, for a synthesis of the work undertaken thus far to accurately identify the biomechanical variables that differ between men and women during landing manoeuvres.

The purpose of this review was to (1) synthesise the reported sex-based differences in lower-limb mechanics of landing manoeuvres; and (2) critically discuss the impact of this body of literature on our understanding of the aetiology of noncontact ACL injuries. First, we summarise research targeting sex-based differences in explicit biomechanical variables during high-risk landings, whilst simultaneously providing rationale for targeting these variables. Second, we address methodological heterogeneity and concerns of this body of literature and discuss their role in the results of our synthesis. Third, we recommend future research directions that aim to more effectively determine why some individuals, especially women, may be more susceptible to noncontact ACL injury than others.

## Sex-based differences in landing mechanics: evidence in the literature?

Whilst a large number of factors have been investigated for sex-specificity pertaining to landing mechanics, the present review focuses on biomechanical variables that have been shown to directly contribute to ACL injury risk (Table 1). These variables have been identified through cadaveric and/or computer modelling studies, during which specific forces are applied to the knee joint (e.g., anterior tibial shear force, tibial internal rotation moment) and resulting ACL loading is measured [15-17]. They have also been identified by observational studies that reported lower-limb joint motion during noncontact ACL injuries [18,19] and by a prospective study that evaluated the knee biomechanical profiles of young girls that went on to rupture their ACL [20].

**Table 1:** Landing kinematic and kinetic variables considered within the current review*

Joint	Kinematic Variables	Kinetic Variables
Knee	Flexion angle	Flexion moment†
	Abduction angle	Abduction moment†
	Internal rotation angle	Internal rotation moment†
	Anterior translation	
Hip	Flexion angle	Flexion moment†
	Adduction angle	Adduction moment†
	Internal rotation angle	Internal rotation moment†

* Variables were chosen based on previously demonstrated links to ACL injury risk

† External moments

To select the research articles that presented original data on sex-based differences in knee and hip mechanics during landing manoeuvres, a literature search was performed. The PubMed database was searched using the following keywords: (1) "sex" OR "gender"; AND (2) "biomechanics"; AND (3) "land*" OR "jump*". Only the articles that met the following criteria, however, were included in the review: (1) healthy, human subjects; (2) comparison of the pre-determined knee and/or hip mechanical variables between sexes, as listed in Table 1; (3) analysis of landing manoeuvres that comprised primarily of a vertical jump or drop jump (and not a landing from a horizontal jump); (4) original research. In addition, the reference lists of included articles were searched for relevant articles that also met the inclusion criteria aforementioned. Thirty-one articles were found and included in the review [8-14,21-44].

### Sagittal plane

Potential links between sagittal-plane knee mechanics and ACL injury are highly touted. This is because the ACL is the primary restraint to anterior tibial translation [45]. In fact, the anterior tibial shear force, which drives this motion, is the most direct way of loading the ACL, particularly when the knee is near full extension [15]. The general line of thinking is that the anterior tibial shear force produced at initial impact during the landing phase translates the tibia anteriorly coinciding with an increase in ACL strain, and thus injury risk. By means of the patella tendon, the quadriceps can produce an anterior tibial shear force, again being largest when the knee is near full extension [46,47]. High quadriceps activation in combination with a small knee flexion angle during a landing is thus viewed as a worst-case scenario for sagittal plane-based ACL injury. An extended hip joint, coupling with the knee joint posture during landings [48], has also been linked to greater anterior tibial shear forces during a landing task [12]. This shear force reported by investigators in this research area, however, must be interpreted with caution. Inconsistencies exist in its definition in the literature (i.e., internal joint reaction shear force vs. external joint shear force), which largely affect its interpretation [49,50]. Recent findings further question the role of anterior tibial shear forces obtained by means of inverse dynamics in estimating ACL loading. It was reported that large shear forces during drop landings do not coincide with high anterior tibial translations, contrary to popular beliefs [49]. Based on the above relations, an examination of knee and hip flexion angles and moments, as well as anterior tibial translation, appears warranted.

A lack of consistency exists in the literature regarding sex differences in knee flexion angles during landings, whilst knee flexion moment data appear to be similar between sexes. Results from most studies indicate that women land with similar knee flexion angles to men [9,10,21,32,33,35-37,42,44]. Several studies, however, did report that women land with a more extended knee than men [8,22,29]. Interestingly, others have observed the opposite outcome [24] (Table 2). With regard to knee flexion moments, consensus among studies suggests that no sex differences exist during landings (Table 2) [10,22,32,36]. Of the two studies to report such differences, however, one found that they were not present across all jump heights tested [40]. Specifically, greater knee flexion moments were evident in female compared to male volleyball players when landing from a 60-cm platform, but not from a 40-cm platform. Further work may be needed into potential height-specific variations in male and female knee flexion moments during landings. Regardless, the body of evidence currently examined does not support the hypothesis that women are at greater risk of ACL rupture during landing tasks due to an extended knee posture.

**Table 2:** Summary of studies investigating sex differences in knee flexion angles and moments during a vertical landing task

References	Population		Landing Task		Result†		
	Category	Mean Age (years)	Double-Leg vs. Single Leg	Height (cm)	at IC	peak	RoM
**Knee Flexion Angle**							
Ford et al. [26]	Adolescent	12-16 (range)	Double	31	0	+	**---**
Walsh et al. [44]	Adult-Athlete	19.5	Double	30.5	0	0	**---**
Salci et al. [40]	Adult-Athlete	21.4	Double	40 & 60	**---**	- (40) 0 (60)	**---**
Hughes & Watkins [29]	Adult-Athlete	21.4	Double	not specified^‡^	-	0	+
Hughes et al. [31]	Adult-Athlete	21.4	Double	not specified^‡^	0	+	+
Russell et al. [39]	Adolescent & Adult	9.5 (youth) 23.9 (adult)	Double	50% of maximum vertical jump height	0	**---**	**---**
Cortes et al. [21]	Adult	23.8	Double	30	0	0	**---**
Chaudhari et al. [9]	Adult	19.9	Double	30.48	0	**---**	**---**
Earl et al. [23]	Adult	22.8	Double	31	**---**	0	**---**
Huston et al. [8]	Adult	28.0	Double	20, 40 & 60	0 (20) - (40 & 60)	0	0
Shultz et al. [12]	Adult	22.4	Double	45	0	**---**	+
Gehring et al. [27]	Adult	23.8	Double	52	0	+	**---**
Decker et al. [22]	Adult	27.5	Double	60	-	**---**	-
Kernozek et al. [32]	Adult	24.1	Double	60	0	0	0
Pappas et al. [37]	Adult	28.5	Double & Single	40	0	0	**---**
Kiriyama et al. [33]	Adolescent	17.0	Single	20	**---**	0	**---**
Lephart et al. [34]	Adult-Athlete	20.3	Single	20	**---**	-	**---**
Fagenbaum & Darling [24]	Adult-Athlete	not specified	Single	25.4 & 50.8	+	+	**---**
Nagano et al. [35]	Adult-Athlete	19.6	Single	30	0	0^a^	**---**
Orishimo et al. [36]	Adult-Athlete	26.3	Single	30	0	0	0
Urabe et al. [42]	Adult-Athlete	22.1	Single	maximum vertical jump height	0	**---**	**---**
Schmitz et al. [41]	Adult	23.2	Single	30	0	**---**	-
Kernozek et al. [10]	Adult	23.4	Single	50	**---**	0	**---**
TOTAL*				**% (-/+/0)**	**20/5/75**	**11/22/67**	**25/38/38**
**Knee Flexion Moment**							
Ford et al. [26]	Adolescent	12-16 (range)	Double	31	**---**	0	**---**
Salci et al. [40]	Adult-Athlete	21.4	Double	40 & 60	**---**	0 (40) + (60)	**---**
Chaudhari et al. [9]	Adult	19.9	Double	30.48	**---**	0	**---**
Shultz et al. [12]	Adult	22.4	Double	45	**---**	+	**---**
Decker et al. [22]	Adult	27.5	Double	60	**---**	0	**---**
Kernozek et al. [32]	Adult	24.1	Double	60	**---**	0	**---**
Orishimo et al. [36]	Adult-Athlete	26.3	Single	30	0	0	**---**
Kernozek et al. [10]	Adult	23.4	Single	50	**---**	0	**---**
TOTAL*				**% (-/+/0)**	**0/0/100**	**0/22/78**	**---**

† +: greater value in female subjects; -: smaller value in female subjects; 0: no differences between sexes; ---: not investigated

^‡ ^Landing from a jump to block a volleyball in the presence of a net of standard, sex-specific height

^a ^knee flexion angle at peak vertical ground reaction force

* Percentage of studies having found lower (-), greater (+), and similar (0) values in female subjects in comparison with male subjects

IC: initial ground contact; RoM: range of motion

Findings regarding sex-based differences in anterior tibial translation appear inconclusive. Given that accurately measuring tibial translations *in vivo *is challenging, only one study has attempted to do so, finding no sex differences [35]. This outcome, however, may be biased by limitations inherent to the methodological approach. Specifically, tibial translation was quantified by means of retro-reflective markers affixed to the skin tracked by a motion capture system. Although a technique suggested to minimise calculation errors due to skin movement artefact was utilised [51], its success in providing accurate joint rotational and translational data has been questioned [52]. This appears especially true for the small range of sagittal-plane tibial translation (≈ 2 mm) evident during landings [53], for which movement artefact error likely dominates the outcome [54,55]. By means of the accurate method of biplane fluoroscopy, Torry et al. [53] did acquire similar data, both in terms of the absence of sex-based differences and magnitude of tibial translations. The participants of this study, however, were instructed to land with limited flexion at the hip, knee and ankle joints. These instructions most likely modified the knee mechanics typically adopted by women and men, making this study of limited use for comparison purposes.

As with knee flexion, evidence of sex differences in hip flexion angles is inconclusive, with no apparent differences in hip flexion moments. The majority of studies revealed no significant sex differences in hip flexion angles during vertical landing tasks [21,22,32,34,36]. For instance, 88% (7 out of 8 studies) reported no sex differences in hip flexion angle at initial ground contact--a point in time when lower-leg posture is critical as most noncontact ACL ruptures occur milliseconds post-contact [19]. Some studies, however, reported either lower or greater hip flexion angles in females compared to males [10,26,29] (Table 3). As for hip flexion moment, no significant differences between sexes were found for the five studies that investigated this dependent variable [10,22,32,36,40] (Table 3).

**Table 3:** Summary of studies investigating sex differences in hip flexion angles and moments during a vertical landing task

References	Population		Landing Task		Result†		
	Category	Mean Age (years)	Double-Leg vs. Single Leg	Height (cm)	at IC	peak	RoM
**Hip Flexion Angle**							
Ford et al. [26]	Adolescent	12-16	Double	31	-	0	---
Salci et al. [40]	Adult-Athlete	21.4	Double	40 & 60	---	- (40) 0 (60)	---
Hughes & Watkins [29]	Adult-Athlete	21.4	Double	not specified^‡^	0	+	+
Cortes et al. [21]	Adult	23.8	Double	30	0	0^a^	---
Shultz et al. [12]	Adult	22.4	Double	45	0	---	+
Decker et al. [22]	Adult	27.5	Double	60	0	---	0
Kernozek et al. [32]	Adult	24.1	Double	60	0	0	0
Lephart et al. [34]	Adult-Athlete	20.3	Single	20	---	0	---
Orishimo et al. [36]	Adult-Athlete	26.3	Single	30	0	0^a^	0
Schmitz et al. [41]	Adult	23.2	Single	30	0	---	-
Kernozek et al. [10]	Adult	23.4	Single	50	---	+	---
TOTAL*				**% (-/+/0)**	**13/0/88**	**11/22/67**	**17/33/50**
**Hip Flexion Moment**							
Salci et al. [40]	Adult-Athlete	21.4	Double	40 & 60	---	0	---
Decker et al. [22]	Adult	27.5	Double	60	---	0	---
Kernozek et al. [32]	Adult	24.1	Double	60	---	0	---
Orishimo et al. [36]	Adult-Athlete	26.3	Single	30	0	0	---
Kernozek et al. [10]	Adult	23.4	Single	50	---	0	---
TOTAL*				**% (-/+/0)**	**0/0/100**	**0/0/100**	**---**

† +: greater value in female subjects; -: smaller value in female subjects; 0: no differences between sexes; ---: not investigated

^‡ ^Landing from a jump to block a volleyball in the presence of a net of standard, sex-specific height

^a ^hip flexion angle at peak knee flexion angle

* Percentage of studies having found lower (-), greater (+), and similar (0) values in female subjects in comparison with male subjects

IC: initial ground contact; RoM: range of motion

### Frontal plane

Frontal-plane knee biomechanics during landings have been investigated extensively as a potential contributor to ACL injury aetiology. This research has been driven primarily by the role of the ACL in restraining motions and loads in this plane, in combination with recent prospective work linking them to injury [15,20]. Markolf et al., for example, found in a cadaveric model that ACL loading increased in the presence of an externally applied knee abduction moment, presenting as a worst-case scenario when combined with an anterior tibial shear force or an internal tibial rotation moment [15]. Also, the work of Hewett and associates [20] demonstrated a prospective link between injury and increased knee abduction angles and moments during landings in young adolescent females [20]. Recent studies have shown that increased knee abduction angles may arise via a transfer of altered hip kinematics during landings, and in particular increased hip adduction angles [56]. Hence, frontal-plane knee and hip mechanics during landings have been compared repetitively between males and females to gain insight into the mechanism of ACL injuries and the sex-biased injury rates.

Evidence in the literature supports greater knee abduction angles in females, whilst that for sex-based differences in frontal-plane moments is inconclusive. Most studies (72%; 13 out of 18 studies) found greater knee abduction angles in females during landings compared to males for at least one variable associated with the ground contact phase (angle at initial contact, peak angle, or total range of motion) [10,11,13,23,25,27,28,30-32,37,38,44] (Table 4). Specifically, females were observed to land with greater peak knee abduction angles in the majority of the studies (71%; 12 out of 17 studies). Most investigations (64%; 7 out of 11 studies), however, did not find sex-based knee abduction differences at initial ground contact [21,25,30-32,35,36]. One could argue that the initial contact abduction angle is more critical to ACL injury risk than the peak angle or range of motion. It is this initial landing posture that governs the frontal plane moment arm magnitude and the resultant external abduction moment, which is known to load the ACL [15]. Also, the knee abduction angle peaks well after the time when ACL injuries are viewed to occur (17-50 milliseconds after initial ground contact [19]). The peak angle, however, may represent a lack of ability in females to control frontal-plane motion of the knee joint. In response to valgus perturbations at the knee, women have shown diminished reflex muscle activation responses in comparison to men [57]. With regards to knee abduction moments during landing manoeuvres, a lack of consistency exists. Whilst 33% of studies (2 out of 6) did find women landed with greater moments [13,14] than men, for example, 17% (1 study) found the exact opposite [32], with the majority (3 studies) reporting no sex differences [9,10,36] (Table 4). Frontal-plane knee mechanics during landings, therefore, may in fact play a role in sex-specific injury risk.

**Table 4:** Summary of studies investigating sex differences in knee abduction angles and moments during a vertical landing task

References	Population		Landing Task		Result†		
	Category	Mean Age (years)	Double-Leg vs. Single Leg	Height (cm)	at IC	peak	RoM
**Knee Abduction Angle**							
Schmitz et al. [11]	Adolescent	14.1	Double	30	---	---	+^a^
Ford et al. [25]	Adolescent	16.0	Double	31	0	+	---
Ford et al. [13]	Adolescent	12-16	Double	31	---	+^b^	---
Hewett et al. [28]	Adolescent	14.1	Double	31	+	+	---
Walsh et al. [44]	Adult-Athlete	19.5	Double	30.5	+	+	---
Hughes et al. [30]	Adult-Athlete	21.4	Double	not specified^‡^	0	+	+
Hughes et al. [31]	Adult-Athlete	21.4	Double	not specified^‡^	0	+	+
Wallace et al. [43]	Adult-Athlete	21.6	Double	maximum vertical jump height	---	0	---
Cortes et al. [21]	Adult	23.8	Double	30	0	0^c^	---
Earl et al. [23]	Adult	22.8	Double	31	---	+	---
Gehring et al. [27]	Adult	23.8	Double	52	+	+	---
Kernozek et al. [32]	Adult	24.1	Double	60	0	+	+
Pappas et al. [37]	Adult	28.5	Double & Single	40	---	+^d^	---
Kiriyama et al. [33]	Adolescent	17.0	Single	20	---	0	---
Nagano et al. [35]	Adult-Athlete	19.6	Single	30	0	0^e^	---
Orishimo et al. [36]	Adult-Athlete	26.3	Single	30	0	0^c^	0
Kernozek et al. [10]	Adult	23.4	Single	50	---	+	---
Russell et al. [38]	Adult	22.5	Single	60	+	+^c^	---
TOTAL*				**% (-/+/0)**	**0/36/64**	**0/71/29**	**0/80/20**
**Knee Abduction Moment**							
Ford et al. [13]	Adolescent	12-16	Double	31	---	+^b^	---
Sigward et al. [14]	Adolescent	14.7	Double	36	---	+^f^	---
Chaudhari et al. [9]	Adult	19.9	Double	30.48	---	0	---
Kernozek et al. [32]	Adult	24.1	Double	60	---	-	---
Orishimo et al. [36]	Adult-Athlete	26.3	Single	30	0	0	---
Kernozek et al. [10]	Adult	23.4	Single	50	---	0	---
TOTAL*				**% (-/+/0)**	**0/0/100**	**17/33/50**	**---**

† +: greater value in female subjects; -: smaller value in female subjects; 0: no differences between sexes; ---: not investigated

^‡ ^Landing from a jump to block a volleyball in the presence of a net of standard, sex-specific height

^a ^frontal-plane angle in two dimensions

^b ^in post-pubertal group only (and not pre-pubertal group)

^c ^knee abduction angle at peak knee flexion angle

^d ^knee abduction angle at 40° knee flexion angle

^e ^knee abduction angle at peak vertical ground reaction force

^f ^average knee abduction moment

* Percentage of studies having found lower (-), greater (+), and similar (0) values in female subjects in comparison with male subjects

IC: initial ground contact; RoM: range of motion

With regard to frontal-plane hip mechanics, the literature does not provide convincing evidence of sex-dependence (Table 5). Differences between men and women were not observed, for example, in hip adduction angle at initial ground contact during drop jump landings [32,36]. Similar outcomes were evident for peak hip adduction angles, with 71% of the studies (5 out of 7) reported no sex-based differences [10,32,34,36,37]. The remaining studies (29%) found women to land with a greater peak hip adduction angles than their male counterparts [23,43]. In addition, all studies investigating hip adduction moments during landing tasks found no sex differences [9,32,36]. Hence, the sex disparity in ACL injury rates does not appear to be explained by sex differences in frontal-plane hip mechanics in landing tasks.

**Table 5:** Summary of studies investigating sex differences in hip adduction angles and moments during a vertical landing task

References	Population		Landing Task		Result†		
	Category	Mean Age (years)	Double-Leg vs. Single Leg	Height (cm)	at IC	peak	RoM
**Hip Adduction Angle**							
Wallace et al. [43]	Adult-Athlete	21.6	Double	maximum vertical jump height	---	+	---
Earl et al. [23]	Adult	22.8	Double	31	---	+	---
Kernozek et al. [32]	Adult	24.1	Double	60	0	0	0
Pappas et al. [37]	Adult	28.5	Double & Single	40	---	0^b^	---
Lephart et al. [34]	Adult-Athlete	20.3	Single	20	---	0	---
Orishimo et al. [36]	Adult-Athlete	26.3	Single	30	0	0^a^	0
Kernozek et al. [10]	Adult	23.4	Single	50	---	0	---
TOTAL*				**% (-/+/0)**	**0/0/100**	**0/29/71**	**0/0/100**
**Hip Adduction Moment**							
Chaudhari et al. [9]	Adult	19.9	Double	30.48	---	0	---
Kernozek et al. [32]	Adult	24.1	Double	60	---	0	---
Orishimo et al. [36]	Adult-Athlete	26.3	Single	30	0	0	---
TOTAL*				**% (-/+/0)**	**0/0/100**	**0/0/100**	**---**

† +: greater value in female subjects; -: smaller value in female subjects; 0: no differences between sexes; ---: not investigated

^a ^hip adduction angle at peak knee flexion angle

^b ^hip adduction angle at 40° knee flexion angle

* Percentage of studies having found lower (-), greater (+), and similar (0) values in female subjects in comparison with male subjects

IC: initial ground contact; RoM: range of motion

### Transverse plane

Although transverse-plane mechanics have received less attention in the literature, they have still been theorised to play an important role in the mechanism of ACL injuries. Krosshaug and colleagues used video footage to assess the knee and hip motions of 30 individuals suffering noncontact ACL injuries. They found that in the majority of cases, the knee medially collapsed with combined hip internal rotation, knee abduction, and tibial *external *rotation [19]. Acknowledging the limitations of estimating joint kinematics and the timing of injury from ordinary television footage, the authors recognised that the tibial *external *rotation may have occurred after ligament rupture. In fact, recent cadaveric injury simulations support this tenet, observing *internal *rotation of the tibia before ligament rupture and *external *rotation following ligament failure [17]. Whilst debate exists with regard to which rotation (internal vs. external) is more risky, most evidence supports tibial internal rotation as the primary contributing factor. Markolf et al. [15], for instance, found that a tibial *internal *rotation moment in combination with an anterior tibial shear force applied to an extended knee produced the greatest ACL force. In contrast, the addition of a tibial *external *rotation moment to an anterior tibial shear force reduced ACL force. Most recently, the application of a tibial *internal *moment was reported to increase ACL strain in comparison to an *external *moment or to an absence of an axial moment [58]. Lower-limb transverse-plane joint mechanics evident during landings, and particularly tibial *internal *rotation, therefore, appear to play an important role in ACL loading and resultant ACL injury risk.

There is limited agreement regarding sex-based differences in transverse-plane knee and hip mechanics during landings (Table 6). Although no sex-based differences were found in tibial internal rotation at initial ground contact [35], greater peak tibial rotations were reported in women for 50% of the studies (2 out of 4) [33,35]. The remaining 50%, however, observed no sex differences [23,34]. A similar trend is present for hip internal rotation angles (Table 6). On the other hand, women and men appear to demonstrate similar knee and hip internal rotation moment magnitudes during landings. It should be noted, however, that only one study to date has investigated these kinetic variables [9].

**Table 6:** Summary of studies investigating sex differences in knee and hip internal rotation angles and moments during a vertical landing task

References	Population		Landing Task		Result†		
	Category	Mean Age (years)	Double-Leg vs. Single Leg	Height (cm)	at IC	peak	RoM
**Knee Internal Rotation Angle**							
Earl et al. [23]	Adult	22.8	Double	31	---	0	---
Kiriyama et al. [33]	Adolescent	17.0	Single	20	---	+	---
Lephart et al. [34]	Adult-Athlete	20.3	Single	20	---	0	---
Nagano et al. [35]	Adult-Athlete	19.6	Single	30	0	+^a^	---
TOTAL*				**% (-/+/0)**	**0/0/100**	**0/50/50**	**---**
**Knee Internal Rotation Moment**							
Chaudhari et al. [9]	Adult	19.9	Double	30.48	---	0	---
**Hip Internal Rotation Angle**							
Earl et al. [23]	Adult	22.8	Double	31	---	0	---
Lephart et al. [34]	Adult-Athlete	20.3	Single	20	---	+	---
**Hip Internal Rotation Moment**							
Chaudhari et al. [9]	Adult	19.9	Double	30.48	---	0	---

† +: greater value in female subjects; -: smaller value in female subjects; 0: no differences between sexes; ---: not investigated

^a ^knee internal/external rotation angle at peak vertical ground reaction force

* Percentage of studies having found lower (-), greater (+), and similar (0) values in female subjects in comparison with male subjects

IC: initial ground contact; RoM: range of motion

In spite of strong assertions regarding "known" biomechanical risk factors for ACL injury, there is limited evidence to suggest that sex-based differences in ACL injury rates arise from concomitant differences in such factors. The one exception is peak knee abduction angle, where women were found to typically land with greater abduction than men. For most biomechanical variables of interest, however, no consensus among studies was reached. Further, for knee and hip flexion angles, and hip adduction angle, the majority of the evidence in the literature supports a lack of sex-based differences.

## Methodological heterogeneity and concerns

When a lack of consistency exists between the results of various studies, investigators have often pointed to methodological discrepancies as the primary cause. The studies investigating sex-based joint mechanical differences within a vertical landing task indeed demonstrate methodological variation in factors such as landing type (single- vs. double-leg landing), height of landing, and population characteristics (adolescents vs. adults, recreational vs. elite athletes). These variations, however, do not appear to govern the lack of consensus observed in most of the variables examined herein. As seen in Tables 2, 3, 4, 5, 6, results did not generally diverge according to the nature of the task or sample population characteristics. Several interesting trends are present, however, indicating that some sex differences may be task- or population-dependent.

First, all of the studies that reported smaller initial contact knee flexion angles in females compared to males examined double-leg landings; whereas the lone study that revealed greater angles investigated a single-leg landing task. This task-dependency is somewhat counterintuitive seeing that single-leg landings appear to be more difficult to execute [37]. During a double-leg landing, both lower-limbs contribute to the deceleration of the centre of mass. It has been suggested, therefore, that a more extended knee posture may be needed to successfully land on a single leg to limit the impact-induced knee flexion moment counteracted by the quadriceps. Given that women are known to have weaker thigh musculature [34], we would expect them to land with an even more extended knee during single-leg landing to minimise this moment. With a stronger musculature, men can afford to perform a single-leg landing with greater initial contact knee flexion angle. Hence, there does not appear to be any logical trend in initial contact knee flexion angle across landing types.

Second, sex differences in frontal- and transverse-plane knee and hip kinematics may also be task-dependent. Both studies that reported greater hip adduction angles in women than men investigated the mechanics of double-leg landings, as oppose to single-leg landings. The increased sensitivity of out-of-plane knee loading to the female 3D hip posture during landings compared to males is well documented [59]. It could thus well be that women have greater difficulty controlling hip motions during double-leg landings. While plausible, evidence supporting this theory of task dependency in frontal-plane hip motion is weak. Only half of the studies assessing hip adduction angles during double-leg landings found women to have greater angles [23,43]. The other studies reported no sex differences [32,37].

Third, greater knee and hip internal rotation angles were present in women during a single-leg landing, whereas double-leg landings did not reveal sex differences in transverse-plane knee and hip mechanics. Given the more challenging nature of the single-leg landing, as stated above, it is plausible that women have a decreased ability to counteract transverse-plane moments produced upon landing, thus leading to greater motion in this plane. In fact, Wojtys et al. [60] have shown women to produce less muscular resistance to internal rotation loading of the tibia, indicative of lower muscular protection of their knee joints.

Lastly, studies reporting greater knee abduction moments in the female group tested adolescent populations, while studies reporting lower female moments or no sex differences tested adults. It is unclear whether an interaction between developmental stage and sex-based knee joint mechanical differences truly exists, or whether it is a coincidence. Only six studies [9,10,13,14,32,36] evaluated sex differences in knee joint abduction moments, and only two tested an adolescent population [13,14]. Across four maturation groups (pre-pubertal, pubertal, post-pubertal, and young adulthood), Sigward et al. [14] found no such interaction. On the other hand, Ford and colleagues [13] reported greater knee abduction moments in post-pubertal girls than boys, whilst no such sex-based differences were observed in the pubertal group. No group of adults, however, were included in their study. Consequently, although a lack of consistency in sex-based knee and hip joint biomechanical differences across studies has often been attributed to concurrent task and age discrepancies, there is limited hard evidence to support such effects.

Some of the discrepancies in results between studies may also stem from anatomical and/or hormonal variations among the participants. None of the studies investigated herein, for example, accounted for potential differences in knee morphology and laxity between participants. Merely two out of the 31 studies controlled for phase of the menstrual cycle during which data collection occurred [9,12]. These factors should not be ignored as they have been shown to influence landing mechanics and ACL injury risk, as well as to be sex-dependent. Knee anatomy, for example, has been reported to differ between ACL-injured patients and uninjured individuals [61-64]. Links have also been found between explicit knee anatomical indices (e.g., posterior-directed slope of the tibial plateau, ACL cross-sectional area) and landing biomechanics (e.g., peak knee abduction, peak knee internal rotation angle, ACL strain) [65-67]. Furthermore, several recent investigations [68,69] consistently support correlations between knee and generalised joint laxity and landing biomechanics, with laxity being dependent on sex [70] and, in women, on menstrual cycle [69,71]. Variations in these additional factors, therefore, may be equally critical to resultant landing biomechanics, thus confounding possible sex-based joint biomechanical differences. Consideration of such factors should be made in future research efforts.

The lack of sex-based differences in knee and hip joint landing biomechanics reported in most studies may be in part due to the means by which joint mechanics are quantified. Typically, joint kinematics are derived from the coordinates (2D or 3D) of retro-reflective markers affixed to the skin that estimate the position of the underlying bones. Whilst skin movement artefact is known to transpire into relatively large errors in the calculation of joint mechanics [54], however, such effects are often disregarded. This can be extremely problematic since errors between 2.2 and 13.1 degrees have been reported in joint rotations for various gait tasks [55,72]. Further, these errors are largest in the frontal and transverse planes, questioning the reliability of sex-based kinematic comparisons in these planes. The validity of these skin marker-derived rotations is thus highly questionable. Given that joint kinetics, such as moments, are also based on data obtained from skin markers, error propagates to these variables as well. Alternatively, dynamic biplane fluoroscopy and x-ray systems offer an accuracy greater than 0.5° for joint rotations and 0.5 mm for joint translations [73]. Such methods, however, afford a limited capture volume, expose participants to radiation, and do not lend well to en-masse testing due to extensive processing time. Despite these shortcomings, future research efforts using such technology as a means toward vast improvements in data accuracy is encouraged.

In addition to error caused by skin movement artefact, data processing techniques are also a common source of error in both kinematic and kinetic data. Overzealous filtering of marker trajectory data may remove critical peaks in joint kinematics and resultant kinetic data. This is particularly problematic if the magnitude of such peaks differs between males and females. Furthermore, discrepancies in kinematic model definitions will also be problematic. Although standards do exist in the biomechanics community [74,75], a wide variety of models are implemented. With regard to joint kinetics, no standards currently exist. This leads to difficulties in reliably comparing both kinematic and kinetic variables across studies. Consequently, collaborative efforts should be made to develop and encourage the adoption of standards with regards to data reduction and processing techniques.

The heterogeneity in the methodologies, the questionable validity of skin marker-based joint mechanics, and potential variations in data processing techniques among studies appears to only partly explain the inconsistencies in reported sex-based joint biomechanical differences. Although some differences appear to be task- or population-dependent, as noted, these trends are generally weak and thus do not seem to explain the lack of consensus in the literature. Also, despite much lower relative error in skin marker-derived joint mechanics in the sagittal plane, evidence of a sex-bias in these examined variables was also inconclusive. Hence, the direct application of the findings of these *in vivo*, laboratory-based studies to elucidate the aetiology of noncontact ACL injury appears to be limited.

Although it was our intention to include all original research that compared knee and hip landing mechanics between healthy females and males, our ability to do so successfully was limited in several ways. As discussed, the studies included in our review demonstrated a diverse range of methodologies, which may have masked commonalities regarding true sex-based differences. Also, we did not systematically quantify the studies' quality. Specifically, we did not report on factors such as adopted methodological standards and techniques, degree of subject proficiency in movement tasks examined, and population sample sizes. Consequently, every study was weighted equally within the review regardless of quality, which may have distorted outcomes. Despite these shortcomings, it is our opinion that our review provides an accurate synthesis of the literature pertaining to sex-based differences in knee and hip landing mechanics.

## Moving beyond the status quo

While studies comparing landing mechanics between male and females have provided key baseline information regarding the ACL injury phenomenon, their ability to speak to the aetiology of these injuries may be limited due to the inherent complexity of the injury mechanism. Given that such studies rarely translate findings to the actual ligament load response *in vivo*, for example, we are faced with the challenge of distinguishing which factors truly manifest within the mechanism from those that merely demonstrate sex-dependence. Measurement of the *in vivo *ligament response during such tasks, via implantable gauges similar to those used by Fleming et al. [76,77] and Cerulli et al. [78], may provide valuable knowledge and should be explored further. Critical insights into sex-specific injury causality have also recently been gained through the linking of explicit knee joint mechanical profiles and resultant ACL loading. Mizuno et al. [79], for example, found that sex-based differences in ACL loading arise even when the same external load states are applied to male and female knee joints. They suggested that differences in joint and ligament morphology, rather than mechanical profile, governed sex-biased injury rates. Further, ACL strain measures were found to be higher when female-specific loading patterns were applied to a cadaveric knee simulating a vertical stop-jump task, in comparison with male-specific patterns [80]. "Stiff" landings, characterised by greater knee and hip flexion, also produced greater ACL force than "soft" landings within a musculoskeletal model [81]. In addition, ACL strain was shown to increase by 30% when a knee abduction moment was added to knee compression and flexion loads in a cadaveric simulation of a single-leg landing [82]. These studies highlight that whilst *in vivo *joint biomechanical data in isolation may not elucidate sex-specific injury risk, they are vital in driving studies that can do so. Specifically, they afford valid input data (e.g., cadaveric and computer model simulations) for estimating ACL loading in response to explicit sex-based neuromechanical scenarios. Success of such studies is still dependent on the accuracy with which these biomechanical inputs can be collected. With this in mind, methods geared towards accurate and reliable *in vivo *joint biomechanical data should be actively pursued.

In light of that presented above, we contend that greater insight into the role of joint mechanics within sex-specific injury risk may be obtained by extending analyses beyond the *in vivo *laboratory setting. Such studies for example, do not adequately replicate the mechanical and neuromuscular control strategies typically utilised within the inherently random field setting where injuries do occur. Myers et al. [73] have suggested that within the laboratory environment, subjects can more effectively modify their muscle activation strategies to ensure a safe movement outcomes when presented with "riskier" movement requirements (i.e., extended knee and hip and greater vertical ground reaction forces (GRF)). Such adaptations may not be possible in the field setting, when additional time and movement constraints prevail. It is additionally plausible that noncontact ACL injuries are due, in part, to a neuromuscular "misfire" that occurs infrequently. If this is the case, then neuromechanical assessments derived from the typically limited number of landing trials (e.g., five to ten) are likely insufficient to capture such a rare event. Validated dynamic models of high-risk landing postures can circumvent some of these issues. The impact of systematic and/or random adjustments in the joint mechanical profile on resultant ACL loading can be readily evaluated [83-85]. Innovative body-worn sensor technologies may also allow us to capture lower-limb kinematics within a more realistic movement environment. Miniature, wireless inertial measurement units (IMUs) [86], for example, could be used directly for this purpose, affording ongoing assessments during relevant, unanticipated athletic manoeuvres during gameplay. Research on the aetiology of noncontact ACL injuries and its sex disparity would benefit, therefore, from studies aimed at either replicating, or direct collection within, naturalistic movement and resultant injury scenarios.

Although evidence in the literature supports a multi-planar injury mechanism, most research efforts continue to be aimed at uni-planar injury risk. This is problematic since the ACL injury mechanism is increasingly viewed to arise via a 3D mechanical mechanism [87]. In the sagittal plane, for example, it is unlikely that the force produced by quadriceps activation is the sole contributor to injury. Specifically, it has been shown that in this plane, the rapid deceleration associated with the landing phase creates a posteriorly directed force vector at the shoe-ground interface, which is transferred to the tibia, protecting the ACL [50,88,89]. Due to the moment balance in the sagittal plane following ground contact, increased quadriceps force, therefore, will be countered by the concomitant increase in the posteriorly directed GRF, promoting minimal changes in the net ACL load [90,91]. In addition, bone bruises consequential of noncontact ACL injuries are commonly found on the posterolateral tibial plateau and lateral femoral condyle [92-94]. This suggests an injury mechanism that combines anterior shear, lateral compression and axial rotation components. An isolated frontal-plane injury mechanism is also unlikely. Within a knee model, an abduction moment applied to the knee joint during a simulated single-leg landing was found to increase ACL strain, but not enough to cause rupture [16]. Knee abduction observed during injurious scenarios may also be a consequence of ligament rupture, and not the inciting event. A cadaveric investigation, for example, reported knee abduction only after ACL failure, and not before failure [17]. Future research efforts, therefore, need to consider the injury mechanism as a likely result of forces and moments produced in a combination of planes.

## Conclusion

This review revealed that there is limited agreement in the literature as to whether women land differently than their male counterparts. Further, when consensus was reached, a lack of sex differences was reported. The only common difference revealed was that women landed with greater peak knee abduction angles than men. Since knee abduction is known to increase ACL loading, therefore, it is plausible that this variable indeed contributes to observed sex-dimorphic injury rates. Conversely, it may simply demonstrate sex-dependence that is irrelevant to the sex disparity in injury risk. To advance our knowledge of the aetiology of noncontact ACL injuries, as a step toward effective injury screening and prevention, we recommend progressing beyond a sex comparison focus. We recommended adopting a focus that includes additional risk factors and assessments within more realistic and less predictable movement environments. Further utilisation of experimental and modelling techniques that afford quantification of the actual ligament response to realistic joint mechanical inputs also seems imperative. Given that joint biomechanical data measured *in vivo *drive these models, these data, however, should not be discounted. In fact, their accuracy is imperative, which fuels the need for the development of superior methods to quantify biomechanics. Unless these critical steps are taken, progress toward elucidating the aetiology of noncontact ACL injuries will be limited.

## Competing interests

The authors declare that they have no competing interests.

## Authors' contributions

MLB performed the literature search and summary. MLB and SGM drafted and edited the manuscript, as well as read and approved its final version.
